# A Case Report of Patella Cubiti: A Rare Diagnosis

**DOI:** 10.7759/cureus.63474

**Published:** 2024-06-29

**Authors:** Issam Berraj, Yassine Saadi, Youness Moudoud

**Affiliations:** 1 Orthopedics and Traumatology, Ibn Sina Hospital, Rabat, MAR

**Keywords:** radiography, sesamoid bone, congenital anomaly, olecranon, elbow

## Abstract

This report describes the case of a 32-year-old patient admitted to the emergency room for polytrauma following a suicide attempt. During the clinical and radiographic examination of the right elbow, Patella cubiti (PC) was fortuitously discovered. This rare anatomical anomaly raises questions about its origin and its impact on the patient's clinical presentation. Despite the generally asymptomatic nature of PC, its association with severe polytrauma in this case underlines the importance of comprehensive radiographic evaluation when managing traumatic injuries. This clinical case contributes to our understanding of this rare anatomical variation and underscores the need for further research on its clinical implications as well as therapeutic management

## Introduction

Patella cubiti (PC) is a very rare condition in which the olecranon, partially or completely, remains separated from the rest of the ulna. It was initially described by Habbe in 1942 [1]. Other terms used to refer to this accessory bone include "olecranon epiphyseal bone" given by Kjelland in 1945 [2] and "sesamum cubiti" described by Sachs and Degenshein in 1920 [3]. PC is more frequent in men, usually unilateral, but bilateral cases also exist [3]. It results from the partial or complete detachment of the olecranon and generally appears on an X-ray as an abnormal bone above the proximal ulna. This abnormal bone most often presents with smooth edges and cortical delineation [4]. We report here the case of a patient admitted for trauma to both heels, in whom PC was fortuitously discovered.

## Case presentation

The patient is a 32-year-old man with a psychiatric condition. He is on antipsychotic medications and was admitted to the emergency department of Ibn Sina Hospital in Rabat following a fall from the second floor in a suicide attempt. The impact point was primarily the lower limbs, resulting in a fracture of the right calcaneus.

During the physical examination, a firm, palpable swelling was discovered at the right elbow, with neither the patient nor his family reporting any prior trauma. The mass, although asymptomatic, had never been examined or diagnosed before. The patient does not have significant limitations in elbow movement, with complete extension and 134° flexion.

The patient was admitted to the emergency department following his fall. The swelling at the right elbow was noted during the initial examination, and X-rays were performed to evaluate its origin.

**Figure 1:**
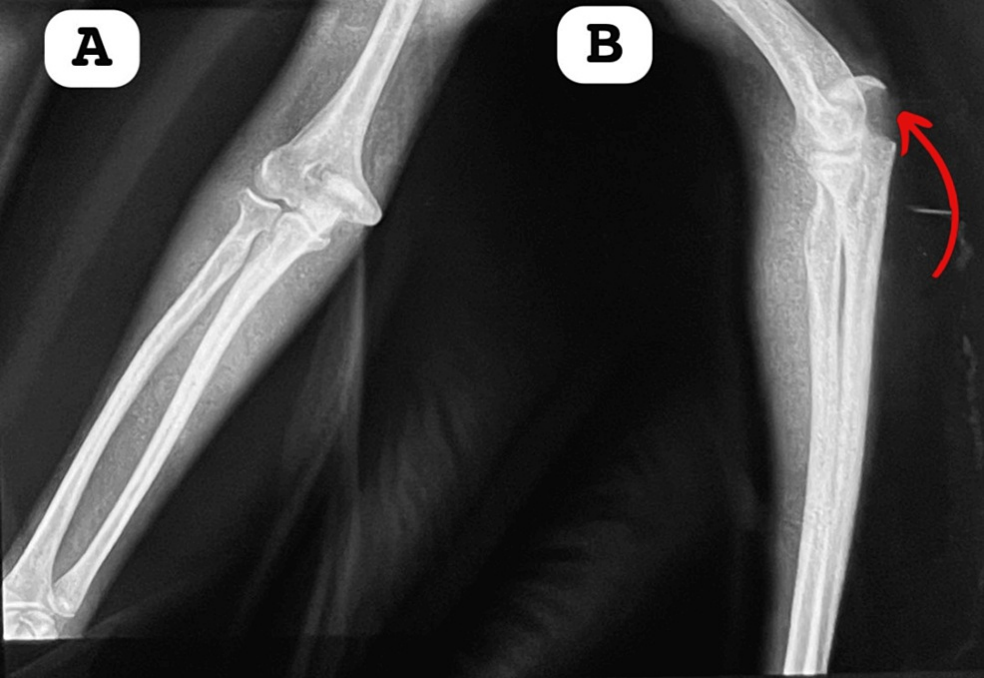
Frontal and lateral X-ray of the patient's right elbow. A: This anteroposterior (AP) radiograph of the elbow shows patella cubiti, an uncommon ossicle, located within the triceps tendon. The image clearly illustrates a distinct bony fragment posterior to the distal humerus. B: This lateral radiograph of the elbow demonstrates the presence of patella cubiti. The image reveals a well-defined ossicle situated within the triceps tendon, visible posterior to the distal humerus and aligning with the elbow joint (indicated by the arrow).

X-rays of the right elbow, in both frontal and lateral views (Figure 1), revealed a bone fragment with regular edges located at the olecranon. X-rays of the left elbow and spine showed no abnormalities. Given the absence of disabling symptoms, no further investigations were deemed necessary.

Due to the absence of symptoms affecting the patient's daily life, a surgical intervention was not performed. Instead, a conservative approach was adopted, consisting of annual clinical and radiological follow-ups to monitor any potential evolution or complications. The patient underwent surgery for his calcaneus fracture.

The annual follow-up includes a clinical evaluation to detect any changes in elbow mobility or the onset of symptoms, as well as X-rays to observe any progression or displacement of the bone fragment. To date, no functional limitations or problematic evolution has been observed.

With regular clinical and radiological follow-ups, the patient can continue his daily life without immediate therapeutic intervention. If symptoms or complications arise, appropriate reassessment and treatment will be considered. The outlook is therefore essentially conservative, with continuous vigilance.

The patient has freely and informedly given his consent for the realization and publication of this manuscript.

## Discussion

Although PC was first described over 80 years ago by Habbe [1], its etiology remains poorly understood. Several hypotheses have been proposed, dominated by congenital, developmental, and traumatic origins.

The congenital hypothesis suggests that the proximal part of the olecranon does not fuse with the proximal ulna during embryonic development, resulting in children being born with PC [5]. The developmental hypothesis proposes that epiphyseal disturbances occurring during early childhood lead to the separation of the olecranon above the metaphyseal plate, resulting in subsequent ossification and the development of PC [6]. The traumatic hypothesis suggests that traumatic separation of the olecranon from the ulna, if untreated, can lead to the formation of PC [7].

The treatment of PC generally depends on the presence of symptoms and the severity of the condition. If the PC is asymptomatic and not causing discomfort, it can simply be observed. In such cases, no active treatment is necessary, but regular follow-up with X-rays may be recommended to monitor the condition's evolution. For patients experiencing elbow stiffness or limited extension, physical therapy may be helpful. Mobilization and strengthening exercises, under the supervision of a physiotherapist, can help improve elbow function [4].

In cases of mild pain associated with PC, over-the-counter pain relievers or anti-inflammatory medications may be prescribed to alleviate symptoms. In severe cases of PC where elbow stiffness is significant and causes substantial discomfort, or when other non-surgical treatments are ineffective, surgical intervention may be considered. Surgery generally aims to remove the abnormal sesamoid bone or correct the condition. Special orthoses or braces may be recommended to maintain the elbow in a specific position, promoting healing or rehabilitation after surgery [8].

The choice of treatment will depend on the physician's evaluation based on the severity of symptoms, the patient's age, physical activity, and the impact of PC on the quality of life [8].

Documented cases are scarce, with the latest case reports from Mittal et al. in 2014 [8] and Winter et al. in 2006 [9]. According to a 2020 anatomical study in the Czech Republic [10], the prevalence of accessory bones, including PC, was less than 0.77% in their sample. This rarity emphasizes the importance of awareness among clinicians to avoid misdiagnosis. Increased reporting and research are crucial to improve understanding and management of this anomaly. Proper recognition can prevent unnecessary interventions and guide appropriate treatment strategies.

## Conclusions

PC is an exceptionally rare condition involving the olecranon's separation from the ulna, typically found incidentally, as demonstrated in our patient. Despite its unusual nature, PC often remains asymptomatic and does not impact daily activities. In this case, the condition was managed conservatively with regular clinical and radiological follow-ups to monitor for any changes. This approach ensures that any potential complications are identified early, allowing for timely intervention if needed. Understanding and recognizing this rare anomaly are crucial for effective patient management.
